# Dynamic scalp topography reveals neural signs just before performance errors

**DOI:** 10.1038/srep12503

**Published:** 2015-08-20

**Authors:** Hiroki Ora, Tatsuhiko Sekiguchi, Yoshihiro Miyake

**Affiliations:** 1 Department of Computational Intelligence and Systems Science, Tokyo Institute of Technology, Yokohama 226-8503, Japan; 2 Honda Research Institute Japan, Wako 351-0193, Japan

## Abstract

Performance errors may cause serious consequences. It has been reported that ongoing activity of the frontal control regions across trials associates with the occurrence of performance errors. However, neural mechanisms that cause performance errors remain largely unknown. In this study, we hypothesized that some neural functions required for correct outcomes are lacking just before performance errors, and to determine this lack of neural function we applied a spatiotemporal analysis to high-density electroencephalogram signals recorded during a visual discrimination task, a d2 test of attention. To our knowledge, this is the first report of a difference in the temporal development of scalp ERP between trials with error, and correct outcomes as seen by topography during the d2 test of attention. We observed differences in the signal potential in the frontal region and then the occipital region between reaction times matched with correct and error outcomes. Our observations suggest that lapses of top-down signals from frontal control regions cause performance errors just after the lapses.

Errors in goal-directed behavior may have serious consequences. For example, while driving a car, if the intention is to stop and the accelerator pedal is mistaken for the brake pedal, a serious traffic accident may result. If neural mechanisms that cause performance errors are identified, we may be able to avoid performance errors by recognizing the error precursor. Therefore, it is important to clarify the mechanisms that cause performance errors.

Electrophysiological studies have provided evidence for a neural mechanism of performance errors. One report speculated that brief lapses in attention, observed as insufficient neural recruiting, were associated with performance errors^1^. Increased negative amplitude was observed in frontocentral scalp sites in trials with error outcomes compared with correct outcomes^1^. The investigators proposed that this phenomenon indicated that attention was reoriented after errors. Other studies found that a positive deviation in response-locked event related potentials (ERPs) was observed in the frontal region in an error preceding a correct trial^2^,^3^,^4^,^5^. These studies suggested that an action–performance monitoring system is associated with performance errors^1^,^2^,^3^,^4^,^5^.

Blood oxygenation level-dependent (BOLD) activity related to performance errors in a system-wide view were reported after functional magnetic resonance imaging (fMRI)^6^. The fMRI study suggested that brief lapses in attention might cause performance errors^6^. This hypothesis is consistent with electrophysiological findings^1^. Another BOLD fMRI study found that an increase of BOLD activity in the default mode network (DMN) correlates with performance errors^7^,^8^,^9^. This correlation suggests that ongoing activity in the frontoparietal control network, which is antagonistic to the DMN, has an important role in maintaining task performance.

Neural mechanisms involving brief lapses in attention have been proposed to cause the performance errors described above. It is thought that frontoparietal cortical areas are responsible for attentional function^10^. These areas may regulate motor and sensory processing in related cortical areas, and this implies that changes in such cortical areas should be observed. Padilla *et al.* reported diminished neuronal recruiting in the visual cortex, and this suggests that inappropriate visual processing caused performance errors^1^. Weissman *et al.* reported higher BOLD activity in the visual cortex in trials with a faster reaction time (RT)^6^. They assumed that there are brief attentional lapses in trials with a relatively slow RT. Therefore, their findings suggest that less BOLD activity in the visual cortex causes performance errors.

Herein we hypothesize that deficiencies in somatosensory processing and/or executive function are associated with performance errors. We focused on ERPs just before performance errors and differences in RTs between the error and correct outcomes of trials. To address the hypothesis, we evaluated differences in dynamic scalp topography of RT-matched ERPs between behavioral task trials with correct and error outcomes. We used RT-matched difference waveforms to overcome the effect of ongoing activity of the frontoparietal control network. To strengthen observations found in previous electrophysiological studies, we used a different behavioral task (d2 test of attention^11^) for our experiments compared with those previously used. The d2 test of attention is widely employed to examine attention and concentration, and was originally developed to measure driving aptitude and efficiency. Thus, we speculated that errors during the d2 test of attention were less artificial than the flanker or Simon tasks because the flanker and Simon tasks produce a response conflict and yield high error rates.

## Results

### Behavioral results

Table 1 shows behavioral results. Because the distribution of RTs was asymmetrical, we adopted the median RT. The median RT was 433–551 ms (group mean of the medians was 463.3 ms). The error rate was 2.0%–13.6% (group mean: 7.3%). “Trials” in Table 1 indicates the number of trials that were not rejected. RTs during trials with error outcomes were shorter than those of trials with correct outcomes (two-tailed paired *t*(9) = 5.36, *p* < 0.001).

### Electrophysiological finding*s*

**Figure 1:**
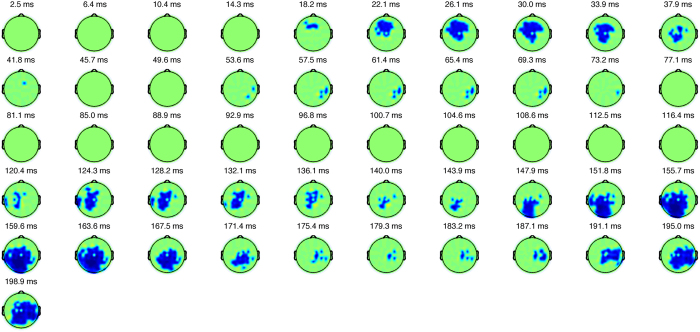
The development in topography of the difference between RT-matched trials with error and correct outcomes. The blue areas indicate statistically significant different amplitudes, and the green areas indicate that there is no statistically significant difference. There are differences in ERPs between RT-matched trials with correct and error outcomes in the frontal region followed by differences in ERPs in the occipital region (FDR corrected).

Figure 1 shows the development in topography of the difference between RT-matched trials with error and correct outcomes. During trials with error outcomes, positive deviation with a latency of approx. 30 ms was observed in frontcentral regions, and then a positive deviation with a latency of approx. 160 ms was observed in the parieto-occipital region (*p* < 0.05, FDR corrected).

**Figure 2:**
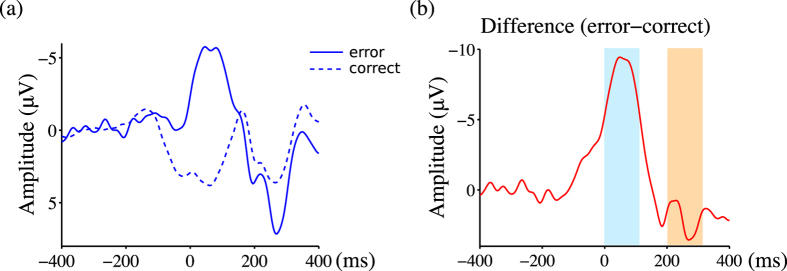
The d2 test of attention elicited error-related potentials. ERPs are response locked. Panels labeled (**a**) show the group mean ERP waveforms at Cz. Solid lines indicate ERP waveforms during trials with error outcomes, while dashed lines indicate ERP waveforms during trials with correct outcomes. Panels labeled (**b**) show the differences between trials with correct and error outcomes. Colored areas correspond to Fig. 4.

**Figure 3:**
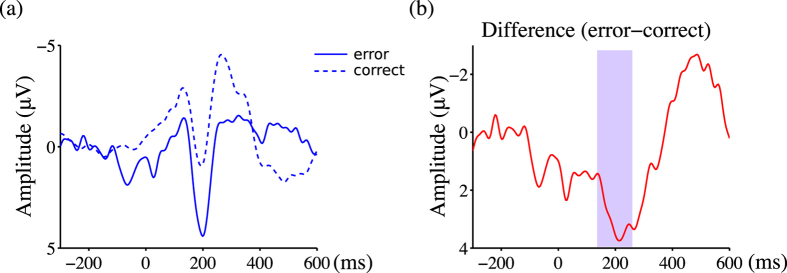
Performance errors in the d2 test of attention modulated the stimulus-locked ERPs. Panels labeled (**a**) show the group mean ERP waveforms at Cz. Solid lines indicate ERP waveforms during trials with error outcomes, while dashed lines indicate ERP waveforms during trials with correct outcomes. Panels labeled (**b**) show the differences between trials with correct and error outcomes. Colored areas correspond to Fig. 4.

**Figure 4:**
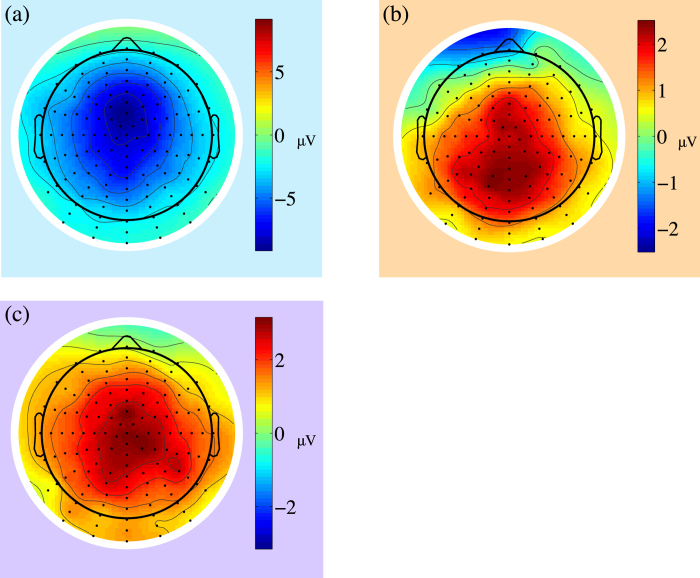
Scalp topographies for difference waveforms (error–correct). Three panels show the difference in scalp ERP topography where the windows were (**a**) 0–100 ms, (**b**) 200–300 ms (response locked) and (**c**) 150–250 ms (stimulus locked). Colors of the regions around the topographies correspond to Figs 2 and 3.

Figures 2, 3 and 4 show the ground averaged waveforms and topological distributions. Figure 2 shows the response-locked group mean waveforms, and Fig. 3 shows the stimulus-locked group mean waveforms. During trials with error outcomes, negative deviation with a latency of less than 100 ms was observed (*t*(9) = –7.56, *p* < 0.001), followed by a positive deviation between 200 and 300 ms (*t*(9) = 4.01, *p* < 0.003) in the response-locked ERPs (Fig. 2). In the stimulus-locked ERPs during trials with error outcomes, a positive deviation between 150 and 250 ms was observed (*t*(9) = 6.99, *p* < 0.001; Fig. 3). During trials with error outcomes, negative deviation between 0 and 100 ms was observed in the parietal region, followed by positive deviation between 200 and 300 ms (Fig. 4).

## Discussion

We evaluated differences in the spatiotemporal pattern of RT-matched ERPs between trials with correct and error outcomes during a d2 test of attention, to examine the hypothesis that a deficit in task-related neural processing causes performance errors. We demonstrated that performance errors in d2 tests of attention modulate stimulus-locked event related potentials, which were largest in the central site. To examine reliability of the phenomena, we selected the d2 test as our behavioral task to contrast with the different tasks used in previous studies. The result of the stimulus-locked ERP analysis shows that there were neural activities before the performance errors that were consistent with previous studies, even though the d2 test of attention was used, and not the flanker task as used in previous studies.

To our knowledge, this is the first report of a difference in the temporal development of scalp ERP between trials with error and correct outcomes as seen by topography during a d2 test of attention. We observed a positive deviation in the frontal region, then in the central region, and then in the occipital region. These results are consistent with those reported by Padilla *et al.*^1^ even though faster RTs during trials with error outcomes are not consistent with those reported by them. This spatiotemporal analysis is considered to reveal sequential development of brain activity across multiple areas before behavioral error.

The latency of the first positive deviation in ERPs was about 50 ms. This indicates that the processing of visual stimuli did not cause this deviation. This deviation might reflect prestimulus brain activity as a result of the prediction of onset of trials with fixed intertrial intervals (3,000 ms). The later positive deviation in the occipital cortex might be associated with enhancement in the visual area that is related to attention, as Padilla *et al.* suggested^1^. Our findings suggest that brief lapses in attention caused performance errors.

What occurs before performance errors? fMRI indicates that an attentional lapse with a slower RT causes performance errors^6^ and this hypothesis has been supported by other findings^1^. It is thought that attention enhances stimuli processing such that RT is shortened. Therefore, it is hypothesized that during trials with slow RTs, attention lapses briefly. However, faster RTs in trials with error outcomes, such as those found in this study, are consistent with findings by other studies^5^,^7^,^12^,^13^,^14^. Therefore, we should also consider the possibility of the existence of trials with a brief lapse in attention along with a fast RT. In such trials, another neural mechanism may cause performance errors. Van Driel *et al.* claimed that there are at least two mechanisms that cause performance errors^15^: inappropriate action impulses and brief lapses in sustained attention. Performance errors, together with faster RTs, suggest that neural events causing brief lapses in sustained attention may cause error. Our findings suggest that both inappropriate action impulses and brief lapses in sustained attention occurred simultaneously in trials with performance errors.

In conclusion, we propose that attentional lapses and inappropriate action impulses cause performance errors. The present study highlights spatiotemporal neural activities through dynamic scalp topography during performance errors. Our findings suggest that deficits in top-down signals from frontal control regions cause subsequent performance errors.

## Methods

### Participants

Ten neurologically normal right-handed adults (average age, 26.6 years old) with normal (7) or corrected-to-normal (3) sight participated in the study. Each participant gave written informed consent before the study and was paid for their participation. The experimental procedures were approved by the Ethics Committee of Honda Research Institute, Japan. All methods were conducted in accordance with the approved guidelines.

### Task

**Figure 5:**
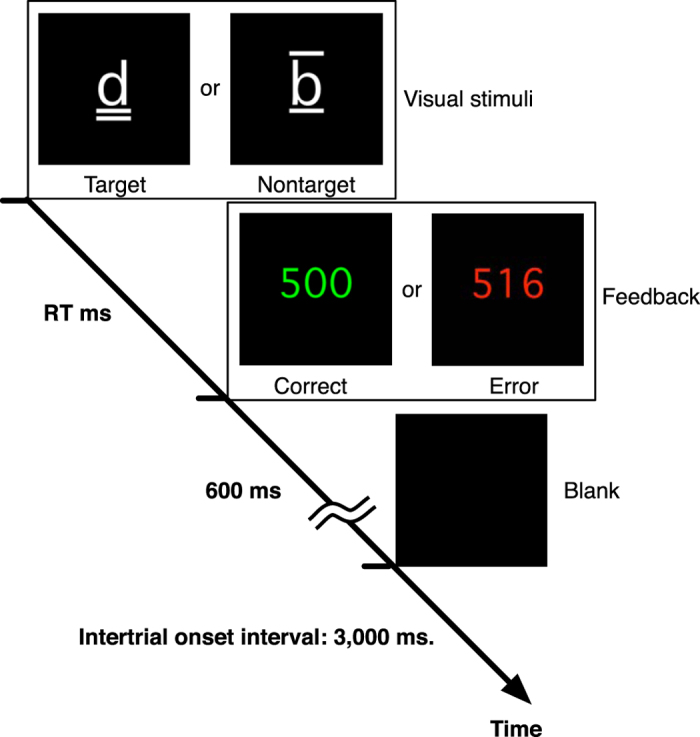
The d2 test of attention. Participants were asked to click the left mouse button when a target was presented and the right mouse button when a nontarget was presented. If the participants made the wrong decision, the outcome was categorized as an “error,” and the RT was presented in red. In the case of “correct” decisions, the RT was presented in green.

Participants sat comfortably in a sound-attenuated chamber 60 cm in front of a visual display. A d2 test of attention^11^ was used (Fig. 5). A trial started with presentation of either target or nontarget visual stimuli. Targets were composited symbols consisting of the character “d” and two dashes that were located both above the “d”, both below the “d” or one above and one below the “d” (Fig. 5. Target). Nontargets were composited symbols consisting of either the character “b” and 0–4 dashes or the character “d” and dashes, whose number differed from two (Fig. 5. Nontarget). Either a target or a nontarget appeared randomly with a probability of 50%. After the visual stimuli were presented on the display, participants had to react to targets by pressing a button with the right index finger, and to nontargets with the right middle finger. Participants were asked to react as quickly and as accurately as possible. Just after participants reacted, a RT was presented on the display for 600 ms, either in green if the reaction was correct (as instructed), or in red if the reaction was erroneous. Then the display was blank until the next trial. The intertrial interval was 3,000 ms. An experimental session consisted of 7–13 runs, and a run consisted of 80 trials. The number of runs for each participant is summarized in Table 1. To stabilize the strategy of the participants for the task, participants performed the task for about 160 trials before the experiment.

### EEG recording

EEG activity was recorded using sintered Ag/AgCl electrodes (128 channels) on the scalp with near uniform distribution using a BioSemi ActiveTwo system (BioSemi, Amsterdam, The Netherlands). The channels were referenced to linked mastoids. To reject blink artifacts, vertical and horizontal electrooculograms were also recorded. The sampling rate for the EEG data was 256 Hz (24-bit depth).

### Data analysis

The stimulus-locked potentials were averaged from 300 ms before to 800 ms after the stimulus, and their baseline was mean amplitude from –300 ms to 0 ms. The response-locked potentials were averaged from –400 ms before to 400 ms after the reaction, and their baseline was mean amplitude from –400 ms to –200 ms. A band-pass filter (3–30 Hz) was applied to the EEG signals. For temporal analysis of the difference between trials with correct outcomes and trials with error outcomes, both RTs were matched, and Student’s *t* statistics were plotted with the *topoplot* function distributed in the EEGLAB^16^ for MATLAB toolbox (Mathworks Inc, Natick, MA, USA). For multiple comparisons correction, we used the false-discovery rate (FDR)^17^ for each channel and time-point. For RT matching between trials with correct and error outcomes, the *ft_stratify* function in the Fieldtrip MATLAB toolbox^18^ was applied in a run-wise manner. To prevent contaminations by artifacts, trials that contained extreme amplitude values (≤–100 μV or ≥100 μV) were rejected from the analysis. Trials that contained a doubled RT, no reaction, or extremely delayed (>1000 ms) reaction, were also rejected from the analysis. To evaluate differences in ERP waveforms between correct trials and error trials, we applied a two-tailed Student’s t test to mean amplitudes within time windows.

## Additional Information

**How to cite this article**: Ora, H. *et al.* Dynamic scalp topography reveals neural signs just before performance errors. *Sci. Rep.* **5**, 12503; doi: 10.1038/srep12503 (2015).

## Figures and Tables

**Table 1:** The behavioral results.

Participant	1	2	3	4	5	6	7	8	9	10
Sex	M	M	M	M	M	M	M	F	M	M
Runs	7	12	12	12	12	13	12	12	12	12
Trials	558	952	958	959	957	1039	949	960	958	940
Median RT (ms)	450	483	433	450	500	483	466	551	400	417
Errors	25	129	97	47	81	21	66	22	120	58
Error rate (%)	4.5	13.6	10.1	4.9	8.5	2.0	7.0	3.4	12.5	6.2

## References

[b1] Padilla M. L., Wood R. A., Hale L. A. & Knight R. T. Lapses in a prefrontal-extrastriate preparatory attention network predict mistakes. J. Cogn. Neurosci. 18, 1477–1487 10.1162/jocn.2006.18.9.1477 (2006). 16989549

[b2] Ridderinkhof K. R., Nieuwenhuis S. & Bashore T. R. Errors are foreshadowed in brain potentials associated with action monitoring in cingulate cortex in humans. Neurosci. Lett. 348, 1–4 10.1016/s0304-3940(03)00566-4 (2003). 12893411

[b3] Allain S., Carbonnell L., Falkenstein M., Burle B. & Vidal F. The modulation of the Ne-like wave on correct responses foreshadows errors. Neurosci. Lett. 372, 161–166 10.1016/j.neulet.2004.09.036 (2004). 15531109

[b4] Hajcak G., Nieuwenhuis S., Ridderinkhof K. R. & Simons R. F. Error-preceding brain activity: Robustness, temporal dynamics, and boundary conditions. Biol. Psychol. 70, 67–78 10.1016/j.biopsycho.2004.12.001 (2005). 16168251

[b5] Eichele H., Juvodden H. T., Ullsperger M. & Eichele T. Mal-adaptation of event-related EEG responses preceding performance errors. Front. hum. neurosci. 4 10.3389/fnhum.2010.00065 (2010). PMC2927308 20740080

[b6] Weissman D. H., Roberts K. C., Visscher K. M. & Woldorff M. G. The neural bases of momentary lapses in attention. Nat. neurosci. 9, 971–978 10.1038/nn1727 (2006). 16767087

[b7] Eichele T. *et al.* Prediction of human errors by maladaptive changes in event-related brain networks. P. Natl. Acad. Sci. USA. 105, 6173–6178 10.1073/pnas.0708965105 (2008). PMC2329680 18427123

[b8] Li C.-S. R., Yan P., Bergquist K. L. & Sinha R. Greater activation of the “default” brain regions predicts stop signal errors. Neuroimage 38, 640–648 10.1016/j.neuroimage.2007.07.021 (2007). 17884586 PMC2097963

[b9] Esterman M., Noonan S. K., Rosenberg M. & DeGutis J. In the Zone or Zoning Out? Tracking Behavioral and Neural Fluctuations During Sustained Attention. Cereb. Cortex 23, 2712–2723 10.1093/cercor/bhs261 (2013). 22941724

[b10] Corbetta M. & Shulman G. L. Control of goal-directed and stimulus-driven attention in the brain. Nat. Rev. Neurosci. 3, 201–215 10.1038/nrn755 (2002). 11994752

[b11] Brickenkamp R. & Zillmer E. D2 Test of Attention. (Hogrefe & Huber, 1998).

[b12] Mazaheri A., Nieuwenhuis I. L. C., Van Dijk H. & Jensen O. Prestimulus Alpha and Mu Activity Predicts Failure to Inhibit Motor Responses. Hum. Brain Mapp. 30, 1791–1800 10.1002/hbm.20763 (2009). 19308934 PMC6870709

[b13] Manly T., Robertson I. H., Galloway M. & Hawkins K. The absent mind: further investigations of sustained attention to response. Neuropsychologia 37, 661–670 (1999). 10390027 10.1016/s0028-3932(98)00127-4

[b14] Robertson I. H., Manly T., Andrade J., Baddeley B. T. & Yiend J. ‘Oops!’: performance correlates of everyday attentional failures in traumatic brain injured and normal subjects. Neuropsychologia 35, 747–758 (1997). 9204482 10.1016/s0028-3932(97)00015-8

[b15] Van Driel J., Ridderinkhof K. R. & Cohen M. X. Not all errors are alike: theta and alpha EEG dynamics relate to differences in error-processing dynamics. J. Neurosci. 32, 16795–16806 10.1523/JNEUROSCI.0802-12.2012 (2012). 23175833 PMC6621791

[b16] Delorme A. & Makeig S. EEGLAB: an open source toolbox for analysis of single-trial EEG dynamics including independent component analysis. J. Neurosci. Meth. 134, 9–21 10.1016/j.jneumeth.2003.10.009 (2004). 15102499

[b17] Benjamini Y. & Hochberg Y. Controlling the false discovery rate: a practical and powerful approach to multiple testing. J. Roy. Stat. Soc. B Met. 57, 289–300 (1995).

[b18] Oostenveld R., Fries P., Maris E. & Schoffelen J. M. FieldTrip: Open source software for advanced analysis of MEG, EEG, and invasive electrophysiological data. Comput. Intell. Neurosci. 2011, 156869 10.1155/2011/156869 (2011). 21253357 PMC3021840

